# Supplementary material for: Alpha-Tocotrienol Prevents Oxidative Stress-Mediated Post-Translational Cleavage of Bcl-xL in Primary Hippocampal Neurons
Source: Int J Mol Sci. 2019 Dec 28;21(1):220. doi: 10.3390/ijms21010220 (PMC6982044; doi:10.3390/ijms21010220)
Supplement: Supplementary file 1 [file ijms-21-00220-s001.pdf]

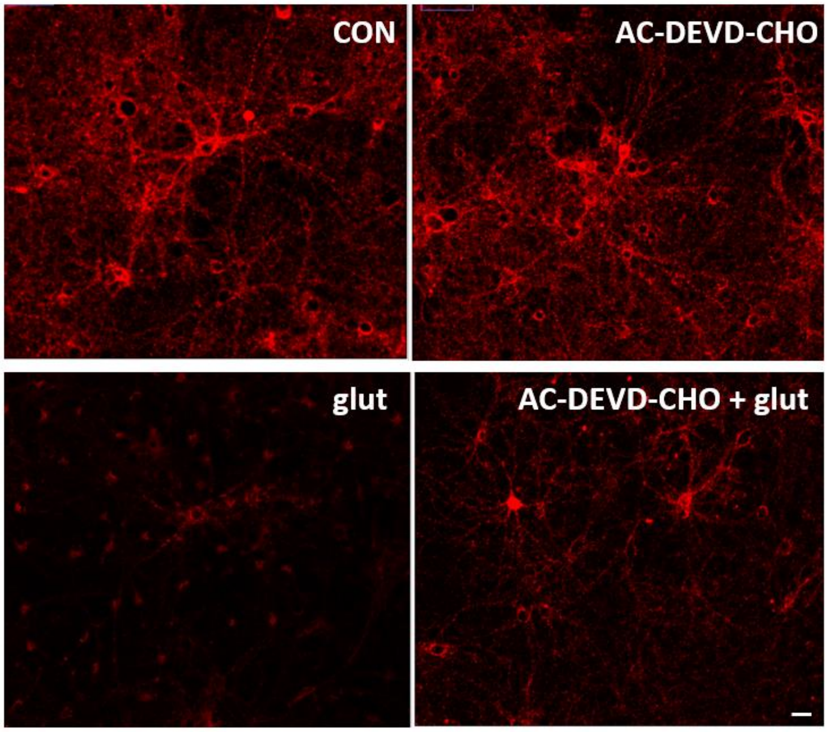

Supplement S1. **AC-DEVD-CHO prevents mitochondrial potential loss against glutamate challenge.** Primary hippocampal neurons were treated with Ac-DEVD-CHO (10μM), glutamate (20μM), or a combination of both. TMRM-stained neurons were imaged at 6h after treatment. Scale bar = 20 μm.
